# Hypertension and dyslipidemia in women with PCOS: a population-based multiregister study in Sweden

**DOI:** 10.1093/humrep/deag064

**Published:** 2026-05-12

**Authors:** S Persson, S Turkmen, A Lindén Hirschberg, I Sundström Poromaa, E Elenis

**Affiliations:** Department of Women’s and Children’s Health, Uppsala University, Uppsala, Sweden; Department of Clinical Sciences, Obstetrics and Gynecology, Sundsvall Research Unit, Umeå University, Umeå, Sweden; Department of Women’s and Children’s Health, Karolinska Institutet, Stockholm, Sweden; Department of Women’s and Children’s Health, Uppsala University, Uppsala, Sweden; Department of Women’s and Children’s Health, Uppsala University, Uppsala, Sweden; Reproduction Centre, Women’s Clinic, Uppsala University Hospital, Uppsala, Sweden

**Keywords:** PCOS, hypertension, dyslipidemia, hyperandrogenism, phenotypes, BMI, multiregister study

## Abstract

**STUDY QUESTION:**

What is the impact of PCOS and the hyperandrogenic (HA) PCOS phenotype on the risk of developing hypertension and dyslipidemia?

**SUMMARY ANSWER:**

PCOS is an independent risk factor for the development of hypertension and dyslipidemia, with the risk being higher among women with the HA PCOS phenotype.

**WHAT IS KNOWN ALREADY:**

PCOS is an established risk factor for insulin resistance and the metabolic syndrome. However, prospective data regarding the risk of hypertension and dyslipidemia in population-based cohorts of women with PCOS are limited.

**STUDY DESIGN, SIZE, DURATION:**

This nationwide multiregister-based cohort study included a total of 297 215 women with PCOS and matched controls followed for up to 20 years. Data were retrieved from the Swedish Patient Register, the Prescribed Drug Register, the Medical Birth Register, the Cause of Death Register, the Total Population Register, and the Education Register.

**PARTICIPANTS/MATERIALS, SETTING, METHODS:**

Study participants resided in Sweden and were born between 1950 and 1999. The median age at study entry was 28 years. Women with a diagnosis of PCOS, androgen excess, or anovulatory infertility recorded in the Swedish Patient Register between 1 January 1997 and 31 December 2016 constituted the study population (n = 50 969). For each woman with PCOS, five controls matched by birth year and municipality were randomly selected from the Total Population Register (n = 246 246). The primary outcomes were incident hypertension and dyslipidemia after PCOS diagnosis, defined by International Classification of Diseases-10 codes and/or filled prescriptions for antihypertensive or lipid-lowering medications, respectively. Women with PCOS were further classified as hyperandrogenic (HA) if they had been diagnosed with androgen excess or had a filled prescription for anti-androgenic drugs in the Prescribed Drug Register; otherwise, they were classified as normoandrogenic (NA). Cox regression analyses were performed, adjusted for birth period, country of birth, and education. Overweight and obesity were accounted for in separate models using either BMI or an obesity ICD-10 diagnosis.

**MAIN RESULTS AND THE ROLE OF CHANCE:**

Women with PCOS had a higher risk of developing hypertension compared with non-PCOS women [adjusted Hazard Ratio (aHR) 2.25 (95% CI: 2.13–2.39), adjusted for obesity]. Those with the HA-PCOS phenotype had more than 5-fold increased risk [aHR 5.51 (95% CI: 4.97–6.10)]. Similarly, PCOS was associated with a higher risk of dyslipidemia [aHR 3.05 (95% CI: 2.69–3.46), adjusted for obesity], and women with the HA phenotype exhibited a more than 7-fold increased risk [aHR 7.82 (95% CI: 6.34–9.64)].

**LIMITATIONS, REASONS FOR CAUTION:**

Inclusion of the most severe cases of PCOS could have led to an overestimation of risk estimates. Information on BMI was only available among parous women. The study comprised mainly women with Nordic origin and should be replicated in cohorts with other ethnicities.

**WIDER IMPLICATIONS OF THE FINDINGS:**

Women with PCOS, especially those with the HA phenotype, face a substantially increased long-term risk of hypertension and dyslipidemia, highlighting the need for early cardiovascular risk assessment and preventive strategies in this population. The findings underscore that PCOS is not only a reproductive disorder but also a significant cardiovascular risk factor, warranting tailored prevention and management strategies.

**STUDY FUNDING/COMPETING INTEREST(S):**

The study was funded by the Family Planning Fund, Uppsala, and the Selanders Fund, Uppsala University (grant number 464251850). Furthermore, E.E. has a part-time research position funded by Uppsala University Hospital (grant number ALF 937815). The funders had no role in the design and conduct of the study; collection, management, analysis, and interpretation of the data; preparation, review, or approval of the manuscript; and decision to submit the manuscript for publication. E.E. has received lecture fees from Merck AB, none in any way related to this manuscript. The rest of the authors declare no conflicts of interest.

**TRIAL REGISTRATION NUMBER:**

n/a.

## Introduction

PCOS is the most common endocrine disorder in reproductive-aged women, affecting up to 10% of them, and is characterized by clinical or biochemical hyperandrogenism, oligo-anovulation, and/or polycystic ovaries (Rotterdam, 2004; March *et al.*, 2010; Bozdag *et al.*, 2016). Hyperandrogenism is identified either biochemically or through the presence of clinical symptoms such as hirsutism, acne, or male pattern hair loss (Rotterdam, 2004; Azziz *et al.*, 2006; Teede *et al.*, 2018). According to the Rotterdam criteria for diagnosing PCOS, which are currently used, at least two features should be present, and other causes should be excluded (Rotterdam, 2004). Four different PCOS phenotypes have been identified based on the combination of these diagnostic criteria, three of which include hyperandrogenism as a feature. Researchers are advised to subdivide women with PCOS into their phenotype groups, if available, and incorporate phenotyping in the statistical analysis (Azziz *et al.*, 2019).

Women with PCOS are commonly insulin resistant with hyperinsulinemia, a feature observed both in lean and obese PCOS women, and these conditions are exacerbated by androgen excess (Barrea *et al.*, 2021). As a consequence, women with PCOS are prone to abdominal obesity, type 2 diabetes, and the metabolic syndrome (Apridonidze *et al.*, 2005; Glintborg, 2016; Lim *et al.*, 2019; Persson *et al.*, 2021). Insulin resistance and hyperandrogenism both contribute to an unfavorable lipid profile by stimulating hepatic lipase activity (Diamanti-Kandarakis *et al.*, 2007). In addition to dyslipidemia, hypertension is also more commonly seen among women with PCOS than in the general female population, with higher blood pressure reported already at a younger age (Elting *et al.*, 2001; Diamanti-Kandarakis *et al.*, 2007; Ollila *et al.*, 2019; Wekker *et al.*, 2020; Zhang *et al.*, 2025). Furthermore, a significantly higher proportion of women with PCOS (50–80%) than non-PCOS women are overweight or obese (Wekker *et al.*, 2020). Therefore, most of the existing studies demonstrating a higher risk for cardiovascular and adverse metabolic outcomes among women with PCOS have focused their interpretation more on the role of obesity and weight gain and less on the PCOS syndrome itself and its phenotypes (Kiconco *et al.*, 2022; Perusquia *et al.*, 2023). The latest ‘*International evidence-based guideline for the assessment of PCOS*’ recommends that all women with PCOS be evaluated for cardiovascular risk factors and blood pressure annually, while all women at diagnosis should undergo a fasting lipid profile (Monash University, 2023). From a clinical point of view, personalizing the advice and surveillance of women with PCOS is reasonable and would contribute to better use of health care resources. Thus, the study objectives were to evaluate the risk of hypertension and dyslipidemia in women with PCOS, after accounting for body weight (BMI or obesity), with particular emphasis on differences between the hyperandrogenic (HA) and normoandrogenic (NA) PCOS phenotypes.

## Materials and methods

### Study design and setting

This was a multi-register population-based matched cohort study. Data were obtained after linkage of six Swedish national registers. The Swedish National Board of Health and Welfare provided data for the Swedish National Patient Register (NPR), the Swedish Prescribed Drug Register (SPDR), the Medical Birth Register (MBR), and the Register on Causes of Death. Statistics Sweden provided data for the Education Register and the Total Population Register (TPR). The study was feasible since all individuals in Sweden are assigned a unique personal identification number upon birth or permanent residency, which enables linkage between registers (Ludvigsson *et al.*, 2009).

The NPR includes information on visiting dates and given diagnoses for inpatient hospital visits since 1964; the information is considered complete since 1987 (Ludvigsson *et al.*, 2011). Since 1997, diagnoses have been classified according to the International Classification of Diseases, version 10 (ICD-10) (Ludvigsson *et al.*, 2011). From 2001 onwards, outpatient hospital visits and visits to specialized healthcare (i.e. private gynecologists) are also included in the register. The MBR contains data on 98% of all births in Sweden since 1973 and includes prospectively collected demographic and clinical data, including information on maternal BMI (Cnattingius *et al.*, 1990; Källén and Källén, 2003). The SPDR contains, since 2005, information on Anatomic Therapeutic Chemical (ATC) classification codes for prescribed drugs, dosage, date of prescription, and date of purchase of the drug (Wallerstedt *et al.*, 2016). The Register of Causes of Death provides information on the date and cause of death. The Education Register contains information on the highest attained level of education of the population. The TPR started in 1968 and provides information on country of birth and municipality of residence (Ludvigsson *et al.*, 2016).

### Study population

All women born between 1950 and 1999 with PCOS (definition follows) were included in the study population. Women diagnosed with PCOS before 12 or after 50 years of age were excluded due to the potential risk of incorrect diagnosis. For each woman with PCOS, up to five non-PCOS women born during the same month and living in the same municipality as the woman in the PCOS group were randomly drawn from the TPR. The index year was defined as the year when a woman was diagnosed with PCOS or included in the study as a matched unexposed individual. Study participants were followed for a maximum of 20 years, with a median follow-up time of 6 years. Median age at study start was 28 years, ranging from 12 to 49 years, and median age at the end of follow-up was 35 years, ranging from 16 to 67 years.

### Exposure

Exposure was defined as having one of the diagnostic codes PCOS (E282), androgen excess (E281), or anovulatory infertility (N970) according to ICD-10 diagnostic codes in the NPR recorded between 1 January 1997 and 31 December 2016. Current literature indicates that ∼75% of women with PCOS have androgen excess (Azziz *et al.*, 2004; Lizneva *et al.*, 2016), while 60–82% of women with hyperandrogenism meet the Rotterdam criteria for PCOS (Carmina *et al.*, 2006; Livadas *et al.*, 2014), rising to 94% in Scandinavian clinical cohorts (Holm *et al.*, 2012). Given the nationwide register-based study design, mainly more severe cases are likely captured, as women with milder phenotypes often remain undiagnosed. To reduce misclassification, we also included anovulatory infertility in the PCOS definition, as ∼80% of anovulatory infertility is attributable to PCOS (Balen *et al.*, 2016). Thus, the presence of androgen excess and anovulatory infertility strongly suggests underlying, potentially undiagnosed PCOS, which motivated their use in defining study exposure. Women with PCOS were further classified according to their PCOS phenotype into NA or HA. Women with androgen excess (E281) registered in the NPR were classified as having the HA phenotype. Further information on hyperandrogenism (clinical or biochemical) at the time of PCOS diagnosis was not available in the registers. Therefore, women with at least two dispensed prescriptions of cyproterone acetate-containing combined oral contraceptives (COCs) (ATC code G03HB01) and/or the antiandrogenic drugs finasteride and eflornithine (ATC code D11AX), finasteride/dutasteride (ATC code G04CB), or flutamide/bicalutamide (ATC code L02BB) at any time during the follow-up in the SPDR were also classified as HA. Since spironolactone is used both in the treatment of cardiovascular disease (CVD) and hypertension, as well as an anti-androgenic medication in women with HA symptoms, all women who were prescribed spironolactone were excluded to minimize potential outcome misclassification and differential selection. COCs are widely used in the PCOS population to treat HA symptoms but are generally associated with a slightly increased risk of hypertension and CVD (Glintborg *et al.*, 2018; Gunaratne *et al.*, 2021). Therefore, they were not included as a proxy for hyperandrogenism.

**Figure 1.**
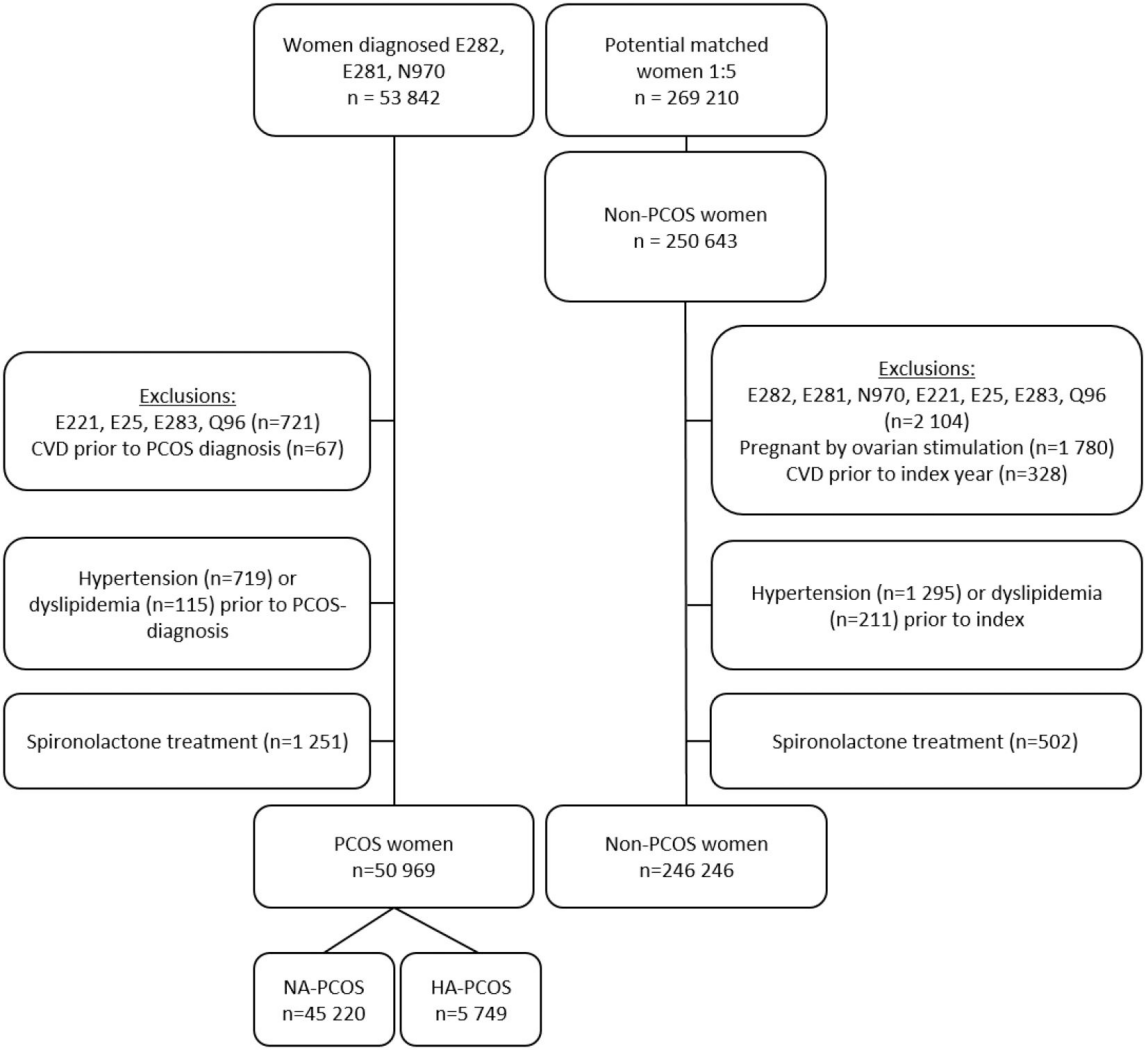
**Flowchart showing study population.** NA-PCOS, normoandrogenic PCOS; HA-PCOS, hyperandrogenic PCOS; CVD, cardiovascular disease. ICD-10 diagnoses: E282, PCOS; E281, androgen excess; N970, anovulatory infertility; E221, hyperprolactinemia; E283, premature ovarian insufficiency; E25, congenital adrenal hyperplasia; Q96, Turner syndrome.

Women in the comparison group with a PCOS diagnosis, anovulatory infertility, or pregnancy achieved by ovarian stimulation were excluded and not replaced. Further exclusion criteria for both groups were diagnosis of hyperprolactinemia (E221), congenital adrenal hyperplasia (E25), premature ovarian insufficiency (E283), and Turner syndrome (Q96). Data on hypothyroidism, hypothalamic amenorrhea, or androgen-secreting tumors were unavailable and therefore could not be excluded. Women diagnosed with CVD, cardiac failure, stroke, hypertension, or dyslipidemia prior to the index date were excluded. After the necessary exclusions, 50 969 women with PCOS and 246 246 matched non-PCOS women were included in the analyses. A flowchart of the study population is shown in Fig. 1.

### Outcomes

The primary outcomes were the development of hypertension or dyslipidemia defined as one of the following: (i) hypertension (ICD-10 code I10) or dyslipidemia (ICD-10 code E78) diagnosis in the NPR or (ii) prescription of antihypertensive or lipid-lowering drugs, according to the SPDR (ATC code C02-C04 or C07-C08 and C10, respectively).

Follow-up started on the index date. Censoring occurred when one of the following occurred: (i) end of follow-up (31 December 2016), (ii) development of hypertension and/or dyslipidemia, or (iii) death.

### Covariates

Information on year of birth was collected from the TPR and categorized into four groups: 1950–1969, 1970–1979, 1980–1989, or 1990–1999. Information on the level of education and the country of birth was collected from the Education Register and the TPR, respectively. We categorized the highest obtained education at the end of the study period as >12 years, 10–12 years, or ≤9 years, and country of birth as being born in the Nordic countries (Sweden, Finland, Denmark, Norway, Iceland), other European countries, the Middle East, South Asia (India, Bangladesh, and Pakistan), Africa, or remaining countries.

Data on BMI and parity were collected from the MBR. Information on height and weight was collected at the first-trimester antenatal visit, and BMI was calculated. In case two or more pregnancies were registered, data from the first pregnancy were used. About half of the women had given birth during the study period, and BMI was available for the majority of them. BMI was categorized as normal- or underweight (<25.0 kg/m^2^), overweight (25.0–29.9 kg/m^2^), or obese (BMI ≥30.0 kg/m^2^). Since a great number of women had no registered childbirth during the study period, and thus lacked information on BMI, we conducted separate analyses adjusting for the ICD-10 diagnosis of obesity (E66) derived from the NPR.

### Statistical analyses

The Statistical Package for the Social Sciences (SPSS) version 28.0.1.0 (142) (SPSS Inc., Chicago, IL, USA) was used for statistical analyses.

### Main analysis

Demographic variables were compared among women with PCOS with NA and HA phenotypes and non-PCOS women. The age at diagnosis of hypertension and dyslipidemia was estimated and compared using one-way ANOVA. We estimated the incidence rates (IRs), adjusted hazard ratios (aHR), and 95% confidence intervals for time to hypertension or dyslipidemia diagnosis using clustered Cox regression, with adjustments for common background confounding factors (i.e. period of birth, country of birth, and education). Since information on BMI was not present in all women, either BMI or obesity diagnosis were entered as confounders, together with the background confounding factors, in two separate models. Model 1 was adjusted for BMI, with a total of 143 374 women included. Model 2 was adjusted for obesity, with a total of 292 446 women included. Furthermore, to compare the risk attributed to body weight as opposed to PCOS status and its phenotypes on the outcomes, we calculated the risk conveyed by the participant’s BMI (i.e. overweight and obese BMI compared to healthy range BMI) or obesity diagnosis, independently of PCOS status, across the entire study population.

### Sensitivity analyses

In order to evaluate the impact of factors associated to the studied exposure or outcomes, we have proceeded with the following sensitivity analyses:

- We explored the effect of the specific ICD-10 diagnoses included in the definition of the exposure by separating the diagnoses of PCOS (E282 and/or E281) and anovulatory infertility (N970).
- Statins (lipid-lowering medications) are recommended as a primary prevention of CVD among individuals with at least one metabolic risk factor (e.g. diabetes, hypertension, smoking) and increased calculated 10-year CVD risk (Bibbins-Domingo *et al.*, 2016). We therefore explored the potential misclassification of the outcome by restricting our dyslipidemia analysis only among individuals with the ICD-10 dyslipidemia diagnosis.
- We studied the effect of time on the categorization of exposure (i.e. PCOS phenotypes). Since our definition of the HA-PCOS phenotype partly relied on the prescription of anti-androgenic drugs and SPDR was first introduced in 2005, we conducted analyses stratified into prior to and after 2005.
- To disentangle the overlapping effects of hyperandrogenism and obesity and enable a direct comparison of their respective risks, we estimated hazard ratios for hypertension and dyslipidemia in non-PCOS women, adjusting for relevant confounders.

### Ethical approval

This study was approved by the Regional Ethical Review Authority in Uppsala, 9 August 2017, diary number 2017/309. Since all data received from the Swedish registries were anonymized, the need for written or oral informed consent was waived.

## Results

Demographic and clinical characteristics of the study population are displayed in Table 1. Almost half of participants were born in the 1980s. About half of non-PCOS women and women with the NA-PCOS phenotype remained childless during the study period, as opposed to 63% of women with the HA-PCOS phenotype. The majority of the study participants were born in the Nordic countries, with a higher proportion of women born in the Middle East among HA-PCOS women. High BMI (≥30 kg/m^2^) and obesity were more prevalent among women with PCOS than among women in the comparison group. The incidences of both hypertension and dyslipidemia were lowest among non-PCOS women (hypertension 1.4% and dyslipidemia 0.2%) and highest in women with the HA-PCOS phenotype (hypertension 7.5% and dyslipidemia 2.0%) (Table 1).

**Table 1.** Demographic and clinical characteristics of the study population.

	Non-PCOS n = 246 246 n (%)	NA-PCOS n = 45 220 n (%)	HA-PCOS n = 5749 n (%)
Birth period			
1950–1969	20 746 (8.4)	4014 (8.9)	265 (4.6)
1970–1979	72 856 (29.6)	13 955 (30.8)	1259 (21.9)
1980–1989	107 097 (43.5)	19 397 (42.9)	2810 (48.9)
1990–1999	45 547 (18.5)	7854 (17.4)	1415 (24.6)
Parity			
No child	121 722 (49.4)	22 387 (49.5)	3637 (63.3)
One child or more	124 524 (50.6)	22 833 (50.5)	2112 (36.7)
Country of birth			
Nordic countries	195 549 (79.4)	34 078 (75.4)	4067 (70.7)
Europe	18 779 (7.6)	3512 (7.8)	442 (7.8)
Middle East	10 843 (4.4)	3624 (8.0)	855 (14.9)
India, Pakistan or Bangladesh	2072 (0.8)	745 (1.6)	64 (1.1)
Africa	6427 (2.6)	997 (2.2)	66 (1.1)
Remaining world	12 576 (5.1)	2264 (5.0)	255 (4.4)
Education			
>12 years	128 632 (52.2)	23 900 (52.9)	3213 (55.9)
10–12 years	92 792 (37.7)	16 721 (37.0)	1992 (34.6)
≤9 years	20 590 (8.4)	4102 (9.0)	504 (8.8)
Missing data	4232 (1.7)	497 (1.1)	40 (0.7)
BMI (kg/m^2^)^1^			
<25.0	80 773 (67.2)	11 227 (51.3)	1048 (51.7)
25.0–29.9	27 355 (22.8)	6054 (27.7)	563 (27.8)
≥30.0	12 020 (10.0)	4614 (21.1)	416 (20.5)
Missing data	126 098 (51.2)	23 325 (51.6)	3722 (64.7)
Obesity diagnosis			
No	235 168 (95.5)	38 587 (85.3)	4941 (85.9)
Yes	11 078 (4.5)	6633 (14.7)	808 (14.1)
Hypertension			
No	242 761 (98.6)	43 670 (96.6)	5319 (92.5)
Yes	3485 (1.4)	1550 (3.4)	430 (7.5)
Dyslipidemia			
No	245 641 (99.8)	44 823 (99.1)	5636 (98.0)
Yes	605 (0.2)	397 (0.9)	113 (2.0)

NA-PCOS, normoandrogenic PCOS phenotype; HA-PCOS, hyperandrogenic PCOS phenotype; BMI, BMI at first antenatal visit of first registered pregnancy.

1 BMI groups presented as valid %, missing data % of total data.

During the follow-up period, a total of 1980 and 510 incident cases of hypertension and dyslipidemia, respectively, were observed, resulting in an IR of 5.91 per 1000 person years [IR 5.91, 95% CI (5.65–6.17)] for hypertension and 1.49 per 1000 person-years [IR 1.49, 95% CI (1.36–1.62)] for dyslipidemia, respectively (Table 2). When the results were stratified by hyperandrogenism, the IR of hypertension and dyslipidemia were 5.17 [IR 5.17, 95% CI (4.91–5.43)] and 1.30 [IR 1.30, 95% CI (1.17–1.43)] in NA-PCOS women and 12.19 [IR 12.19, 95% CI (11.04–13.35)] and 3.09 [IR 3.09, 95% CI (2.52–3.66)] in HA-PCOS women, respectively (Table 3).

**Table 2.** Incidence rates of hypertension and dyslipidemia in non-PCOS vs PCOS women during follow-up.

	Non-PCOS N = 246 246		PCOS N = 50 969		
	N incident cases (%)	Incidence rate per 1000 PY	N incident cases (%)	Incidence rate per 1000 PY	*P*-value
Hypertension	3485 (1.4%)	2.12 (2.05–2.20)	1980 (3.9%)	5.91 (5.65–6.17)	<0.001
Dyslipidemia	605 (0.2%)	0.37 (0.34–0.40)	510 (1%)	1.49 (1.36–1.62)	<0.001

PY, person-years.

**Table 3.** Incidence rates of hypertension and dyslipidemia in non-PCOS vs NA-PCOS and HA-PCOS women during follow-up.

	Non-PCOS N = 246 246		NA-PCOS n = 45 220		HA-PCOS n = 5749		
	N incident cases (%)	Incidence rate per 1000 PY	N incident cases (%)	Incidence rate per 1000 PY	N incident cases (%)	Incidence rate per 1000 PY	*P*-value
Hypertension	3485 (1.4%)	2.12 (2.05–2.20)	1550 (3.4%)	5.17 (4.91–5.43)	430 (7.5%)	12.19 (11.04–13.35)	<0.001
Dyslipidemia	605 (0.2%)	0.37 (0.34–0.40)	397 (0.9%)	1.30 (1.17–1.43)	113 (2.0%)	3.09 (2.52–3.66)	<0.001

NA-PCOS, normoandrogenic PCOS phenotype; HA-PCOS, hyperandrogenic PCOS phenotype; PY, person-years.

### Main findings on hypertension

Overall, women with PCOS had an increased risk of hypertension compared with non-PCOS women, both when the analyses were adjusted for participants’ BMI [Model 1, aHR 2.08 (95% CI: 1.92–2.25)], as well as when adjusted for obesity [(Model 2), aHR 2.25 (95% CI: 2.13–2.39)], Table 4.

**Table 4.** Risk of hypertension and dyslipidemia in non-PCOS vs PCOS women.

	Non-PCOS aHR (95% CI)	PCOS aHR (95% CI)
	Model 1, adjusted for BMI	
	n = 119 545	n = 23 829
Hypertension	(ref)	2.08 (1.92–2.25)
Dyslipidemia	(ref)	2.84 (2.36–3.40)
	Model 2, adjusted for obesity diagnosis	
	n = 242 014	n = 50 432
Hypertension	(ref)	2.25 (2.13–2.39)
Dyslipidemia	(ref)	3.05 (2.69–3.46)

Hazard ratios adjusted (aHR) for birth period, country of birth and educational level. Model 1 adjusted also for BMI, Model 2 adjusted also for obesity diagnosis.

Table 5 displays the risk of developing hypertension and dyslipidemia according to PCOS phenotypes. Women with the HA-PCOS phenotype had a nearly six times higher risk of hypertension compared with women without PCOS, independently of BMI or presence of obesity [Model 1; aHR 5.91 (95% CI: 5.09–6.86) and Model 2; aHR 5.51 (95% CI: 4.97–6.10), respectively]. Women with the NA phenotype had a 2-fold higher risk of hypertension compared with non-PCOS women [Model 1; aHR 1.77 (95% CI: 1.63–1.94) and Model 2; aHR 1.94 (95% CI: 1.83–2.07)].

**Table 5.** Risk of hypertension and dyslipidemia in non-PCOS vs NA-PCOS and HA-PCOS women during follow-up.

	Non-PCOS aHR (95% CI)	NA-PCOS aHR (95% CI)	HA-PCOS aHR (95% CI)
	Model 1, adjusted for BMI		
	n = 119 545	n = 21 803	n = 2026
Hypertension	(ref)	1.77 (1.63–1.94)	5.91 (5.09–6.86)
Dyslipidemia	(ref)	2.47 (2.04–3.00)	7.32 (5.28–10.16)
	Model 2, adjusted for obesity diagnosis		
	n = 242 014	n = 44 723	n = 5709
Hypertension	(ref)	1.94 (1.83–2.07)	5.51 (4.97–6.10)
Dyslipidemia	(ref)	2.62 (2.29–2.99)	7.82 (6.34–9.64)

NA-PCOS, normoandrogenic PCOS phenotype; HA-PCOS, hyperandrogenic PCOS phenotype. Hazard ratios (aHR) adjusted for birth period, country of birth, and level of education. Model 1 adjusted also for BMI, Model 2 adjusted also for obesity diagnosis.

### Main findings on dyslipidemia

In addition, women with PCOS had a 3-fold increased risk of dyslipidemia compared with non-PCOS women, [aHR 2.84 (95% CI: 2.36–3.40) when adjusted for BMI and aHR 3.05 (95% CI: 2.69–3.46) when adjusted for obesity], Table 4. Women with the NA-PCOS phenotype had a 2-fold increased risk for dyslipidemia compared to unexposed women, regardless of BMI or obesity. The risk of dyslipidemia was even higher among women with HA-PCOS compared with non-PCOS women; seven times higher when adjusted for BMI [aHR 7.32 (95% CI: 5.28–10.16)] and eight times when adjusted for obesity [aHR 7.82 (95% CI: 6.34–9.64)] (Table 5).

Lastly, women with the HA-PCOS phenotype were diagnosed with hypertension and dyslipidemia at a lower mean age, 32 ± 7.4 years and 32 ± 7.6 years, compared with NA-PCOS women (35 ± 8.2 and 35 ± 8.3 years) and non-PCOS women (35 ± 8.2 and 35 ± 8.3 years), *P* < 0.001.

### Sensitivity analyses

- Sensitivity analysis was performed after stratifying exposed women to those with PCOS specific diagnosis (E282 and/or E281) or anovulatory infertility (N970) separately, as shown in Supplementary Tables S1 and S2. The estimated HRs for developing hypertension were somewhat lower among women with anovulatory infertility than among women with PCOS-specific diagnoses.
- In the sensitivity analyses where dyslipidemia was solely based on the diagnostic code, NA-PCOS women had an aHR of 2.20 (95% CI: 1.74–2.79) in Model 1, while the corresponding estimate in women with HA-PCOS was aHR 2.67 (95% CI: 1.45–4.92). In Model 2, the hazard ratio was 2.26 (95% CI: 1.93–2.64) for NA-PCOS women and 2.51 (95% CI: 1.71–3.68) for HA-PCOS women, as displayed in Supplementary Table S3. Thus, the estimates restricted to ICD-diagnosed dyslipidemia were similar among NA-PCOS women but were significantly attenuated among HA-PCOS women.
- In the sensitivity analysis examining PCOS phenotypes by timing of PCOS diagnosis (i.e. before or after 2005) in relation to the development of hypertension, the results remained unaltered. However, the risk of dyslipidemia was higher among the HA-PCOS women diagnosed before 2005, particularly after adjustment for obesity (Supplementary Tables S4 and S5).
- When restricting the analysis to non-exposed women (non-PCOS), the aHRs for hypertension and dyslipidemia nearly doubled in overweight women and tripled in obese women—risks that remained lower than those associated with hyperandrogenism and PCOS (Supplementary Table S6).

## Discussion

### Main findings

Overall, this large population-based multi-register study confirms the increased risk of hypertension and dyslipidemia among women with PCOS. A novel finding is that women with the HA-PCOS phenotype have a more than 5-fold increased risk of developing hypertension and a 7-fold higher risk of developing dyslipidemia compared to women without PCOS, independently of their body weight. Both conditions also tend to develop at an earlier age than in the reference group. The estimated risk conveyed by PCOS, and hyperandrogenism in particular, is higher than that of other well-known risk factors, such as obesity, and should therefore be taken into consideration when counseling women with PCOS about future health risks. However, it should be stressed that registers typically capture only the most severe cases of a disease, which could, in turn, exaggerate the risk estimations but would not induce false associations.

### Pathophysiology

The exact pathway linking PCOS and hyperandrogenism to hypertension and dyslipidemia remains unknown, but (central) adiposity is considered a key driver of dysregulated lipid metabolism (Moran *et al.*, 2017). Nonetheless, several other contributing pathways are likely involved. Insulin resistance and hyperinsulinemia, often seen in PCOS, have been suggested to promote lipogenesis, enhance lipolysis, and alter clearance of apo-lipoproteins (Diamanti-Kandarakis *et al.*, 2007; Macut *et al.*, 2008; Cerf, 2013; Liu *et al.*, 2019; Guo *et al.*, 2022), collectively contributing to lipid abnormalities. Androgen excess contributes to these conditions through multiple mechanisms, including exacerbating insulin resistance, promoting visceral fat accumulation, and directly triggering enzymes involved in lipid synthesis (Diamanti-Kandarakis *et al.*, 2007). In fact, clinical hyperandrogenism has a more pronounced effect on lipid profile compared to biochemical hyperandrogenism (Guo *et al.*, 2022). Moreover, evidence suggests that androgen excess may induce epigenetic alterations of genes involved in lipid and steroid synthesis, further driving hyperandrogenism (Liu *et al.*, 2022). Ectopic lipid accumulation is another factor, as it promotes the secretion of proinflammatory cytokines, induces oxidative stress (lipotoxicity), and triggers chronic inflammation. This chronic inflammation, in turn, significantly contributes to hypertension by increasing arterial stiffness and causing endothelial dysfunction (Connelly *et al.*, 2022). Lastly, testosterone increases renin levels, while insulin positively correlates with aldosterone levels, thereby contributing to vasoconstriction and imbalance in the autonomic nervous system mechanisms that further drive hypertension (Connelly *et al.*, 2022; Profili *et al.*, 2024). These findings align with our results, which demonstrate that women with the HA phenotype have a higher likelihood of hypertension and dyslipidemia compared to women without this condition.

### Clinical relevance

The expanded international evidence-based guideline for the management of PCOS recommends that all women with PCOS be assessed for cardiovascular risk factors at each visit, and if this screening reveals any risk factors (i.e. obesity, smoking, dyslipidemia, hypertension, impaired glucose tolerance, or lack of physical activity), the woman should be considered at high risk of developing CVD (Monash University, 2023). Current guidelines recommend that all women with PCOS undergo annual blood pressure measurements, regardless of the presence of other CVD risk factors (Monash University, 2023). Furthermore, fasting lipid profile measurements are recommended to all women with PCOS at diagnosis, independently of age and BMI, with follow-up based on dyslipidemia and global cardiovascular risk factors. Both recommendations are in accordance with our findings. What this study adds to the literature is that it highlights, for the first time, the aggravating role of clinical hyperandrogenism in PCOS and distinguishes it from the risk conveyed by body weight. The markedly increased risk of hypertension—nearly 6-fold—in HA-PCOS women contrasts with the more moderate risk observed in NA women. This suggests that NA women with PCOS may require less intensive hypertension screening, which could reduce the burden on healthcare systems.

### Comparison to other studies

There is a scarcity of studies exploring cardiometabolic diseases and PCOS in the literature. Systematic reviews have reported conflicting findings on hypertension risk in women with PCOS (Millán-de-Meer *et al.*, 2023; Pan, 2023; Pourahmad *et al.*, 2025). Pourahmad *et al.* reported an increased risk for hypertension, independently of BMI, while Pan found no association, suggesting that body weight alone may not fully account for the development of hypertension in women with PCOS. In contrast, Millán-de-Meer *et al.* (2023) suggested that most cardiovascular and metabolic comorbidities are driven primarily by obesity and excess weight—a conclusion not fully supported by our findings. In addition, a Nordic cross-sectional study reported higher BMI and waist circumference, as well as abnormal blood pressure in women with PCOS compared to controls, with BMI showing a positive correlation with androgen levels (Pinola *et al.*, 2017).

Pinola *et al.* (2017) also explored metabolic parameters in relation to PCOS phenotype, such as cholesterol and lipoproteins, and demonstrated more abnormal lipid profile among PCOS women, with the highest low-density lipoprotein and triglyceride levels seen in HA-PCOS women. Similar findings were demonstrated in the review by Cooney and Dokras (2018). A recent large Canadian register study found that women with PCOS developed dyslipidemia at an earlier age and with a higher incidence than controls; however, it lacked detailed information on PCOS symptoms, including hyperandrogenism (Vine *et al.*, 2024).

Lastly, Daan *et al.* (2014) compared the prevalence of hypertension as well as dyslipidemia between the HA- and NA-PCOS phenotypes and concluded that women with the HA phenotype had a higher prevalence of both hypertension and dyslipidemia than women with the NA phenotype, a finding that is in agreement with ours.

### Strengths and limitations

The strengths of our study include its large sample size, the population-based study design spanning a wide time range, and detailed subdivision into PCOS phenotypes. Furthermore, all data were sourced from validated national registers, ensuring comprehensive and reliable coverage.

A limitation of the study was the markedly lower prevalence of hyperandrogenism in our PCOS population compared to the expected in other populations (11% as opposed to 75%) (Lizneva *et al.*, 2016), likely reflecting underdiagnosis and limitations in coding practices. ICD codes could not provide information on the exact diagnostic criteria that were fulfilled at the time of PCOS diagnosis; we could not, therefore, subdivide the PCOS phenotypes with certainty or in a more detailed manner than in just HA or NA phenotypes. Moreover, when defining the HA-PCOS phenotype, we relied on either a recorded diagnosis of androgen excess or the prescription of disease-specific anti-androgenic drugs, both of which underestimate the proportion of HA women. We deliberately did not include additional clinical androgenic features such as hirsutism or acne, which have low specificity as isolated diagnostic markers for PCOS. The diagnostic approach may overlook milder cases of hyperandrogenism, as this diagnosis is not consistently recorded in the registers. Lastly, in the context of potential clinical underreporting or underdocumentation, clinicians may not consistently assign a separate ICD code for androgen excess in women with an existing PCOS diagnosis, further limiting case identification. Likewise, the treatment-based approach may exclude HA women who have not received pharmacological therapy. On the other hand, COCs are the recommended first-line treatment for hyperandrogenism in women with PCOS (Monash University, 2023) but are contraindicated in women with hypertension, and caution should be taken in obese women due to the elevated risk of VTE (Bulletin, 2019). Since COC use is overrepresented among women with HA symptoms and has been linked to hypertension (Yanes Cardozo *et al.*, 2017; Gunaratne *et al.*, 2021), we chose not to include it in our definition of the HA-PCOS phenotype. Further, we lacked data on cyproterone acetate alone and therefore did not include it among the anti-androgenic agents. Lastly, since treatment indication is not available in SDPR, we excluded all women prescribed spironolactone—whether for anti-androgenic or antihypertensive use—as we could not determine whether it represented the exposure or the outcome, which further reduced the study population. We can therefore not rule out that this approach introduced differential misclassification, likely attenuating the positive effect, without inducing spurious associations.

Another limitation was the unavailability of data on the potential presence of androgen-secreting tumors as causes of androgen excess or hypothalamic amenorrhea as causes of anovulatory infertility, all of which, however, are considered rare within their respective categories. In addition, we lacked information on common risk factors for hypertension and dyslipidemia, such as smoking, lifestyle, and physical activity. We were also unable to account for family history for these conditions, which could likewise influence the observed associations. For women who did not give birth during the study period, data on BMI were lacking. Furthermore, women who gave birth at a young age or early during the follow-up period might have a different BMI than when the outcome developed; thus, the BMI recorded might not correctly reflect the metabolic status during the entire study period. We attempted to overcome this limitation by adjusting our analyses for obesity, based on the recorded ICD-10 diagnosis, instead of BMI. We should, though, mention that in a register study performed in Denmark, the validity of obesity diagnosis was reported to be high, but the completeness of diagnosis registered was low (Gribsholt *et al.*, 2019). Most likely, a greater number of (non-PCOS and PCOS) women are obese while lacking a recorded diagnosis in the register. This misclassification would weaken the associations between obesity and outcomes and exacerbate, somewhat, the association with PCOS.

Additionally, general recommendations on CVD prevention encourage physicians to prescribe statins to women aged 40–75 years with at least one CVD risk factor and an estimated 10-year CVD risk of 10% or greater (Mangione *et al.*, 2022). The latter would in turn mean that not all lipid-lowering prescriptions in SPDR necessarily mirror an underlying dyslipidemic disease but could instead be a preventive measure. To overcome this potential source of outcome misclassification, we restricted all analyses on dyslipidemia, including mainly ICD-10 diagnoses of the disease (with/without prescriptions of lipid-lowering medications). The sensitivity analysis revealed similar estimates among NA-PCOS women but markedly higher estimates among HA-PCOS women in the analysis, including lipid-lowering drugs. This suggests that either hyperandrogenism increases the risk of dyslipidemia requiring treatment or that statin use reflects elevated overall cardiovascular risk—rather than dyslipidemia alone—prompting prophylactic treatment for CVD (such as the presence of diabetes, hypertension, or smoking) and thus reflects a higher-risk population.

Lastly, we cannot exclude a risk of misclassification regarding the exposed population and the definition of exposure, a known limitation of register-based studies as opposed to unselected populations (Lizneva *et al.*, 2016; Atiomo *et al.*, 2024). The population of women with PCOS is derived from specialized gynecological outpatient and inpatient care and did not capture primary care, where many women with PCOS-related symptoms initially present. Furthermore, it relies solely on registered diagnoses, indicating that only the most severe cases were included. As a result, our risk estimates may be exaggerated, as they likely reflect HA-PCOS women with severe HA symptoms that were not sufficiently controlled by COCs. Additionally, the oldest women in our comparison group may have undiagnosed PCOS, as most women are diagnosed during their reproductive years, which preceded our register data. In contrast, younger women may not yet have received their PCOS diagnosis during the study period. A sensitivity analysis based on PCOS diagnosis received before or after 2005 (when the SPDR was established) yielded similar results as the main analysis, except for obese women diagnosed with HA-PCOS before 2005. These women likely reflect an older portion of the population that inevitably has more frequent contact with healthcare providers and are therefore more likely to be diagnosed with various conditions.

## Conclusions

Women with PCOS had a moderately increased risk of developing both hypertension and dyslipidemia, regardless of body weight. Specifically, women with the HA-PCOS phenotype had a 6-fold increased risk of developing hypertension and a 7-fold increased risk of developing dyslipidemia compared to women without PCOS, a risk far greater than the one associated with obesity. We therefore propose that the PCOS diagnosis in general, and the HA phenotype in particular, be included as independent risk factors when counseling women on the risk of hypertension, dyslipidemia, and CVD risk. Further clinical trials are warranted to assess whether early screening and/or prophylactic treatment of PCOS women with lipid-lowering drugs, antihypertensives, or anticoagulants can prevent future cardiovascular events like myocardial infarction and stroke.

## Supplementary Material

deag064_Supplementary_Table_S1

deag064_Supplementary_Table_S2

deag064_Supplementary_Table_S3

deag064_Supplementary_Table_S4

deag064_Supplementary_Table_S5

deag064_Supplementary_Table_S6

## Data Availability

Restrictions apply to the availability of the data generated or analyzed during this study because they were used under license. The corresponding author will on request detail the restrictions and any conditions under which access to some data may be provided.
