# Supplementary material for: Hypertension and dyslipidemia in women with PCOS: a population-based multiregister study in Sweden
Source: Hum Reprod. 2026 May 12;41(7):1197–206. doi: 10.1093/humrep/deag064 (PMC13334923; doi:10.1093/humrep/deag064)
Supplement: deag064_Supplementary_Table_S1 [file deag064_supplementary_table_s1.pdf]

Human Reproduction, 2026, 1–1
https://doi.org/10.1093/humrep/deag064
Supplementary Data

human reproduction

OXFORD

Supplementary Table S1. Sensitivity analysis based on PCOS diagnosis; hazard ratios adjusted for BMI.

	Non-PCOS aHR (95% CI)	NA-PCOS aHR (95% CI)	HA-PCOS aHR (95% CI)
N970	n = 37 869	n = 8286	n = 253
Hypertension	(ref)	1.26 (1.06–1.51)	5.42 (3.38–8.70)
Dyslipidemia	(ref)	1.39 (0.92–2.10)	5.64 (1.77–17.97)
E282/E281	n = 82 239	n = 13 609	n = 1774
Hypertension	(ref)	2.07 (1.87–2.29)	6.09 (5.20–7.14)
Dyslipidemia	(ref)	3.08 (2.46–3.86)	7.66 (5.40–10.86)

NA-PCOS, normoandrogenic phenotype; HA-PCOS, hyperandrogenic phenotype. Hazard ratios adjusted (aHR) for birth period, country of birth, educational level, and BMI.
