# Supplementary material for: Hypertension and dyslipidemia in women with PCOS: a population-based multiregister study in Sweden
Source: Hum Reprod. 2026 May 12;41(7):1197–206. doi: 10.1093/humrep/deag064 (PMC13334923; doi:10.1093/humrep/deag064)
Supplement: deag064_Supplementary_Table_S2 [file deag064_supplementary_table_s2.pdf]

Human Reproduction, 2026, 1–1
https://doi.org/10.1093/humrep/deag064
Supplementary Data

human reproduction

OXFORD

Supplementary Table S2. Sensitivity analysis based on PCOS diagnosis; hazard ratios adjusted for obesity.

	Non-PCOS aHR (95% CI)	NA-PCOS aHR (95% CI)	HA-PCOS aHR (95% CI)
N970	n = 72 089	n = 15 861	n = 658
Hypertension	(ref)	1.37 (1.20–1.56)	5.30 (3.61–7.79)
Dyslipidemia	(ref)	1.47 (1.09–1.99)	6.17 (2.71–14.07)
E282/E281	n = 164 157	n = 29 359	n = 5091
Hypertension	(ref)	2.26 (2.10–2.42)	5.68 (5.10–6.33)
Dyslipidemia	(ref)	3.18 (2.74–3.71)	8.18 (6.56–10.20)

NA-PCOS, normoandrogenic PCOS phenotype; HA-PCOS, hyperandrogenic PCOS phenotype. Hazard ratios adjusted (aHR) for birth period, country of birth, educational level, and obesity.
