# Supplementary material for: Hypertension and dyslipidemia in women with PCOS: a population-based multiregister study in Sweden
Source: Hum Reprod. 2026 May 12;41(7):1197–206. doi: 10.1093/humrep/deag064 (PMC13334923; doi:10.1093/humrep/deag064)
Supplement: deag064_Supplementary_Table_S3 [file deag064_supplementary_table_s3.pdf]

Human Reproduction, 2026, 1–1
https://doi.org/10.1093/humrep/deag064
Supplementary Data

human reproduction
OXFORD

Supplementary Table S3. Sensitivity analysis based on dyslipidemia diagnosis E78 only; adjusted hazard ratios among women with PCOS and non-PCOS women.

	Non-PCOS aHR (95% CI)	NA-PCOS aHR (95% CI)	HA-PCOS aHR (95% CI)
Model 1, adjusted for BMI	n = 119 545	n = 21 803	n = 2026
Dyslipidemia	(ref)	2.20 (1.74–2.79)	2.67 (1.45–4.92)
Model 2, adjusted for obesity	n = 242 014	n = 44 723	n = 5709
Dyslipidemia	(ref)	2.27 (1.93–2.64)	2.51 (1.71–3.68)

NA-PCOS, normoandrogenic PCOS phenotype; HA-PCOS, hyperandrogenic PCOS phenotype; BMI, BMI at first antenatal visit of first registered pregnancy. Hazard ratios adjusted (aHR) for birth period, country of birth, educational level, and BMI/obesity. Adjusted hazard ratios among women with PCOS and non-PCOS women.
