# Supplementary material for: Hypertension and dyslipidemia in women with PCOS: a population-based multiregister study in Sweden
Source: Hum Reprod. 2026 May 12;41(7):1197–206. doi: 10.1093/humrep/deag064 (PMC13334923; doi:10.1093/humrep/deag064)
Supplement: deag064_Supplementary_Table_S4 [file deag064_supplementary_table_s4.pdf]

Human Reproduction, 2026, 1–1
https://doi.org/10.1093/humrep/deag064
Supplementary Data

human reproduction

OXFORD

Supplementary Table S4. Sensitivity analysis divided in PCOS diagnosis before or after 2005; hazard ratios adjusted for BMI.

	Non-PCOS aHR (95% CI)	NA-PCOS aHR (95% CI)	HA-PCOS aHR (95% CI)
PCOS before 2005	n = 25 305	n = 5102	n = 384
Hypertension	(ref)	1.73 (1.50–1.98)	5.93 (4.60–7.64)
Dyslipidemia	(ref)	2.64 (1.98–3.51)	8.39 (5.10–13.78)
PCOS after 2005	n = 94 803	n = 16 793	n = 1643
Hypertension	(ref)	1.82 (1.63–2.03)	6.00 (5.00–7.21)
Dyslipidemia	(ref)	2.42 (1.86–3.16)	6.54 (4.22–10.12)

NA-PCOS, normoandrogenic PCOS phenotype; HA-PCOS, hyperandrogenic PCOS phenotype; BMI, BMI at first antenatal visit of first registered pregnancy. Adjusted for birth period, country of birth, educational level, and BMI.
