# Supplementary material for: Hypertension and dyslipidemia in women with PCOS: a population-based multiregister study in Sweden
Source: Hum Reprod. 2026 May 12;41(7):1197–206. doi: 10.1093/humrep/deag064 (PMC13334923; doi:10.1093/humrep/deag064)
Supplement: deag064_Supplementary_Table_S5 [file deag064_supplementary_table_s5.pdf]

Human Reproduction, 2026, 1–1
https://doi.org/10.1093/humrep/deag064
Supplementary Data

human reproduction
OXFORD

Supplementary Table S5. Sensitivity analysis divided in PCOS diagnosis before or after 2005; hazard ratios adjusted for obesity.

	Non-PCOS aHR (95% CI)	NA-PCOS aHR (95% CI)	HA-PCOS aHR (95% CI)
PCOS before 2005	N = 41 404	N = 7888	N = 729
Hypertension	(ref)	1.84 (1.67–2.02)	5.77 (4.85–6.87)
Dyslipidemia	(ref)	2.51 (2.07–3.04)	9.43 (6.94–12.83)
PCOS after 2005	N = 204 842	N = 37 332	N = 5020
Hypertension	(ref)	2.03 (1.87–2.20)	5.46 (4.81–6.20)
Dyslipidemia	(ref)	2.75 (2.29–3.31)	6.79 (5.10–9.05)

NA-PCOS, normoandrogenic PCOS phenotype; HA-PCOS, hyperandrogenic PCOS phenotype; BMI, BMI at first antenatal visit of first registered pregnancy. Adjusted for birth period, country of birth, educational level, and obesity.
