# Supplementary material for: Hypertension and dyslipidemia in women with PCOS: a population-based multiregister study in Sweden
Source: Hum Reprod. 2026 May 12;41(7):1197–206. doi: 10.1093/humrep/deag064 (PMC13334923; doi:10.1093/humrep/deag064)
Supplement: deag064_Supplementary_Table_S6 [file deag064_supplementary_table_s6.pdf]

Human Reproduction, 2026, 1–1
https://doi.org/10.1093/humrep/deag064
Supplementary Data

human reproduction OXFORD

Supplementary Table S6. Risk of hypertension and dyslipidemia in non-PCOS women, normal weight vs overweight and obese.

	**BMI <25.0 aHR (95% CI)**	**BMI 25.0–29.9 aHR (95% CI)**	**BMI ≥ 30.0 aHR (95% CI)**
Hypertension	(ref)	1.46 (1.31–1.63)	2.79 (2.48–3.14)
Dyslipidemia	(ref)	1.87 (1.43–2.44)	2.52 (1.84–3.44)

	No obesity aHR (95% CI)	Obesity aHR (95% CI)
Hypertension	(ref)	3.88 (3.55–4.25)
Dyslipidemia	(ref)	4.87 (3.98–5.97)

Hazard ratios adjusted (aHR) for birth period, country of birth, and educational level.
